# Feasibility and acceptability of strategies to address mental health and mental ill-health in the Australian coal mining industry

**DOI:** 10.1186/s13033-018-0245-8

**Published:** 2018-11-01

**Authors:** Ross J. Tynan, Carole James, Robyn Considine, Jaelea Skehan, Jorgen Gullestrup, Terry J. Lewin, John Wiggers, Brian J. Kelly

**Affiliations:** 1 Everymind, PO Box 833, Newcastle, NSW 2300 Australia; 2 Centre for Resources Health and Safety, NIER, University of Newcastle, 70 Vale Street, Shortland, NSW Australia; 3 School of Medicine and Public Health, University of Newcastle, Hunter Building, University Drive, Callaghan, PO Box 833, Newcastle, NSW 2300 Australia; 4 MATES in Construction, Spring Hill, QLD Australia; 5 Centre for Brain and Mental Health Research, School of Medicine and Public Health, University of Newcastle, Callaghan, PO Box 833, Newcastle, NSW 2300 Australia; 6 Hunter New England Population Health, Booth Building, Longworth Avenue, Wallsend, NSW 2287 Australia

**Keywords:** Workplace health, Mental health, Mining, Coal, Peer-support

## Abstract

**Background:**

To evaluate the feasibility, acceptability and effectiveness of implementing a peer-based, multi-component mental health program in the Australian coal mining industry.

**Methods:**

The multicomponent program included MATES in mining (a peer-based mental health and suicide prevention program) and supervisor training. Eight Australian coal mines participated in the research, with four mines receiving the mental health program. Primary outcome variables including mental health stigma, help-seeking behaviour and perception of the workplace commitment to mental health were measured prior to program implementation, and then again 10 months following using a paper based survey. Process evaluation of the mental health program was measured using a pre-test/post-test survey.

**Results:**

*MATES in mining* 1275 miners participated in the MATES general awareness and connector training. Participants were more confident that they could identify a workmate experiencing mental ill-health; help a workmate, family member or themselves identify where to get support and were more willing to start a conversation with a workmate about their mental health. *Supervisor training* 117 supervisors completed the supervisor training and were subsequently more confident that they could: identify someone experiencing mental ill-health in the workplace; identify and recommend support services to a person experiencing mental ill-health; and have an effective conversation about performance issues that may be due to mental ill-health.

**Conclusions:**

Our findings show evidence to support the use of peer-based mental health programs in the mining industry, and for male-dominated industry more broadly.

## Background

Findings from the most recent Australian National Survey of Mental Health and Wellbeing (NSMHWB) indicate that 45.5% of the Australian population aged between 18 and 65 had experienced mental illness (e.g. depression, anxiety and/or substance use) at some stage in their lives [1]. Mental ill-health is associated with substantial economic burden, with an estimated annual cost to the Australian Government of $20 billion.

Being employed is generally associated with better mental health and wellbeing [2], as well as a range of social and economic benefits [3]. The workplace can provide an important setting for interventions to promote better mental health among employees; support recovery from mental ill-health, and mitigate the risk of mental ill-health by identifying and minimizing workplace hazards [4]. Building and maintaining a mentally healthy workforce and creating a mentally healthy workplace maximises employee wellbeing, and can increase employee productivity [4].

Conversely, unaddressed mental ill-health can have an adverse impact in the work setting. These are most commonly expressed in terms of lost productivity, mental ill-health related absenteeism, presenteeism (attending work, but not working at full capacity), workplace injury and staff turnover [5]. It is estimated that mental health problems incur cost to Australian business of $10.9 billion each year [6]. Importantly, previous research suggests that even simple workplace mental health programs can yield substantial returns on investment [7], with an estimated return of $2.30 for every $1.00 invested in effective action [6].

There is some evidence to indicate higher rates of depression in male dominated industries, when compared to other industries or the general population [8]. However, addressing mental ill-health in male-dominated industry presents a number of challenges, as men tend to have a lower mental health literacy [9], and are less likely to discuss mental ill-health with either professional or non-professional sources of support [10]. Mental illness stigma is also higher in men [11], which is often associated with a number of adverse consequences, including a reluctance to seek help [12, 13]. As a result, mental illness within male-dominated industries may be more likely to go unrecognised and untreated. It is particularly noteworthy that suicide rates are highest in some male-dominated occupations [14], reflecting greater rates of suicide among males in general [15], but also suggesting contributing factors in specific occupational groups (e.g. “blue-collar” occupations) [14].

Evidence suggests that workplace mental health programs are most effective when they adopt a multi-component approach [16, 17], with the determinants of mental ill-health widely recognised to be multi-faceted in nature [18]. Elements of effective workplace mental health programs include the provision of education regarding mental health and mental illness, increasing the capacity of supervisors/managers to identify and respond to mental illness in their staff, having workplace policy that supports employees who have mental health problems, and return to work programs available to support employees transitioning back to work after a period of absence [19].

### Mining and mental health

Mining is one of the largest male-dominated industries in Australia, directly employing an estimated 2% of the total national workforce, with over 100 coal mines operating across the two primary coal producing states: New South Wales (NSW) and Queensland (QLD). Media and community attention on mental health of employees in the mining industry has been triggered by concerns regarding suicide in mining occupations [20].

There are a number of challenges to addressing mental ill-health within the mining industry. Male-dominated industries in general are often characterised as possessing a stoic ‘macho’ culture, which combined with the stigma surrounding mental illness, may present as a significant attitudinal barrier to the overall acceptance of mental health related programs [21, 22]. Other factors associated with working in the mining industry that may impact on employee mental health and wellbeing include: working long hours, compression rosters, shift work, performing tasks that are both physically demanding and repetitive, working considerable distances from home, and displacement from familial social support networks. Most mines are located in rural or remote areas, where the availability of local professional support services is limited [23]. To be effective, mental health programs must overcome these unique cultural and social challenges, and tailor a program to meet the specific needs of the industry. Hence, any program developed for the mining industry needs to be both evidence-based and informed by industry specific needs, to ensure it is culturally relevant and acceptable.

## The “working well” program

Initially a baseline assessment of mental ill-health was conducted, which identified significantly higher alcohol use [24] and levels of psychological distress [25] among mining employees when compared to population prevalence data. The findings also highlighted the preferred patterns for seeking assistance was by consulting with non-professional sources of support for mental health related problems [10], as has previously been reported in other male-dominated long distance commute workforces [26, 27]. These results informed the selection of programs suited to the specific needs of the industry. Peer based programs have been effective in other work settings in promoting and facilitating early help-seeking behaviour [4].

The current study aimed to evaluate the feasibility, acceptability and effectiveness of implementing a peer-based, multi-component mental health program in the Australian coal mining industry. The program core was built on a mining focussed modification to a highly successful peer-based suicide prevention and mental health promotion program developed for the construction industry (“MATES in construction”) [28] which focuses on suicide prevention, promoting awareness of mental ill-health, and providing tools to identify those who may require more specialised support.

## Methods

The study was approved by the University of Newcastle Human Ethics Committee (Approval Numbers: H-2013-0135 and H-2013-0421).

### Study design

The research targeted inclusion of eight mines. Ten mines were approached, with eight mines agreeing to participate in the study. The non-participating sites could not allow sufficient time during the data collection window. Coal mines were selected for Phase One using a quota sampling approach to ensure a representative cross section of the industry. This process involved stratification of the selection criteria, to ensure representative coverage of mine type (3 open cut; 5 underground). Four sites (2 open cut; 2 underground) were selected, using a quota sampling method, to receive the multi-component working well mental health program (WWMHP), with the remaining four sites to serve as ‘comparator’ sites. Sites that participated in the WWMHP completed a pre-test and post-test evaluation on the day of training. Phase Two data occurred 10 months following Phase One at both WWMHP and control sites occurred.

### The working well mental health program (WWMHP)

The WWMHP consisted of a number of complementary components. The core was MATES in mining (MIM), a pilot of the multi-modal mental health and suicide prevention program based on the MATES in Construction program [28]. This consists of three levels of training which are used to establish a peer based ‘gatekeeper’ network on the worksite:

1. All workers on site participate in a one hour ‘general awareness training’ (GAT), which aims to increase awareness of mental illness and suicide, improve knowledge of warning signs, and encourage workers to offer support to colleagues in need.
2. Subsequently, volunteer ‘connectors’ are recruited and provided with an additional four hours of ‘gatekeeper training’, which aims to provide skills on identifying risk and techniques for engaging co-workers.
3. Key workers on site are also provided a two-day ‘applied suicide intervention skills training’ (ASIST), which prepares staff to offer support in a crisis situation.
4. The on-site peer-support program is complemented by field officers, case management support and a 24/7 helpline [29].

This model was chosen for WWMHP, given the success of the program in the construction industry [28–31] which in part is attributed to the fact that presenters have previous experience working in male-dominated industry, which provides a sense of authenticity and credibility.

### Supervisor training

The MIM training was complemented by a tailored two hour skill based training for supervisor and frontline leaders developed for the WWMHP in response to an industry identified need. Supervisor training provided an introduction to mental health and mental ill-health, and used a series of vignette based case-studies to promote discussion of issues relevant to managing staff experiencing mental ill-health and/or suicidal behaviour. The focus was on building confidence, skills and capacity to respond effectively.  

### Process evaluation of the WWMHP

A process evaluation was conducted before and after the MIM and the supervisor training. The purpose of this evaluation was to assess program implementation, acceptability and immediate changes in participant confidence.

### Mates in mining (MIM) and supervisor training

For sites that received the MIM and/or supervisor training, all participants were invited to complete a paper-based pretest/post-test evaluation survey during training. The survey included identical questions across both time points to measure participant change and training effectiveness, with additional questions asked exclusively at post-training that canvassed acceptability, attitudes towards program content and implementation.

### Outcome evaluation

The outcome evaluation was designed to measure longer-term effects of the WWMHP training. This evaluation assessed changes, at a site level, in employee perception of mental illness stigma, help-seeking for mental health related problems, and perception of the workplace commitment to employee mental health. The data were collected over two phases, with Phase One before training, and Phase Two an average of 10 months later.

### Participation and recruitment

All participating mines were sent information packages prior to data collection including posters for display, and PowerPoint slides for presentation at pre-shift meetings. A researcher provided a brief presentation outlining the purpose of the research and distributed information statements and paper based surveys onsite at each data collection point. Surveys, took an average of 15 min to complete. All staff currently working at the participating mine sites at the time of data collection were invited to participate in the study.

### Primary outcome variables

The domains of assessment were guided by the workplace psychosocial safety climate model [32], with all instruments informed by theory, expert review and industry advice supporting rigour and pragmatism.

Key outcome data were collected to assess changes at the site level following implementation of the WWMHP. The primary outcome measures included an evaluation of mental illness stigma, help-seeking behaviour for mental ill-health, and the perception of workplace support for mental health related issues.

### Stigma

The perception of mental health stigma in the workplace was measured using a perceived stigma scale [33]. Participants were asked to indicate whether they felt that an employee would be treated poorly in the workplace if they were to disclose that they had been diagnosed with a mental illness. Perceived stigma was measured on a five-point scale ranging from ‘strongly disagree or disagree’ (low stigma) to ‘agree or strongly agree’ (high stigma).

### Help-seeking

Participants were asked to self-report the number of times they had consulted with various support people to discuss their own mental health within the preceding 12 months, using a previously validated tool [33].

### Perception of workplace commitment to mental health

Using a five-point scale (range: 1—strongly disagree to 5—strongly agree), we assessed various dimensions regarding the perception of workplace support: offering flexible work arrangements; mental health training for employees and supervisors; management understanding of mental health related issues; and workplace policy supporting the mental health of employees.

### Data analysis

Microsoft excel and SPSS (version 23) were used for all data analysis.

WWMHP pretest–posttest evaluation data were analysed descriptively, and using paired sample *t*-tests where appropriate.

Initial analysis of the Phase One and Phase Two data was descriptive in nature, focussing on sample characteristics and prevalence rates of primary outcome variables. Chi square analysis and independent *t*-tests were used where appropriate. All significant Chi square were followed up by a 2 × 2 factorial ANOVA (treatment: comparator vs WWMHP; phase: Phase One vs Phase Two) to explore differences in the magnitude of effect size.

As the research did not preclude new participants at Phase Two, logistic regression was used to determine any significant changes in the composition of the sample between Phase One and Phase Two.

For all statistical analysis, we used an α criterion of 0.01 to partially control for the number of statistical tests.

## Results

### MATES in mining—feasibility, acceptability, and effectiveness

Across the four sites that received the WWMHP, a total of 1163 participants (1014 male; 135 female; 14 not specified) completed the MIM GAT program (site range 86–569). MIM Connector training was completed by 114 participants (92 male; 10 female; 12 not specified).

### Impact of training

Participants who completed either the MIM GAT and/or the connector training displayed significant post-training improvements in their self-reported confidence in identifying workmates with a mental health problem, where they could get support, willingness to start of conversation with a workmate, and perceptions of the workplace as supporting mental health of the site employees (see Table 1).

**Table 1:** Paired sample *t*-test to measure change before and after MIM GAT and connector training

	General awareness training (GAT) (*n *= 1163) Mean (SD)			Connector training (*n *= 114) Mean (SD)		
	Pre	Post	*p*	Pre	Post	*p*
I am confident that I could identify if a workmate was experiencing a mental health problem	3.16 (0.84)	3.57 (0.76)	< 0.001	3.75 (0.82)	4.42 (0.62)	< 0.001
I am willing to start a conversation with a workmate about their mental health or my worries for them	3.44 (1.00)	3.78 (0.86)	< 0.001	3.98 (0.93)	4.65 (0.57)	< 0.001
I am confident that I could help a workmate, family member or myself identify where to start to get support	3.47 (0.94)	3.86 (0.81)	< 0.001	3.97 (0.89)	4.65 (0.59)	< 0.001
I am aware of the structures and systems that my workplace has in place to support worker mental health	3.29 (1.05)	3.68 (0.91)	< 0.001	3.60 (1.02)	4.29 (0.88)	< 0.001
I believe my workplace tries to look after worker mental health	3.24 (1.04)	3.53 (1.05)	< 0.001	3.53 (0.99)	4.08 (1.00)	< 0.001
I believe my workplace does a good job supporting workers experiencing mental health problems	3.23 (1.20)	3.47 (1.03)	< 0.001	3.38 (1.04)	3.96 (1.02)	< 0.001

Scores from 1 (not at all) to 5 (very much)

### Perceptions of training

Participants reported that the training was relevant, useful, well delivered, and is something that they would recommend to others. On a five-point scale (range: 1—not at all to 5—very much so), participants mean scores indicated that they felt that the content was relevant (GAT M = 4.1, SD = 0.8; connector M = 4.7, SD = 0.5), useful (GAT M = 4.3, SD = 0.7; connector M = 4.8, SD = 0.6), satisfactorily delivered (GAT M = 4.4, SD = 0.7; connector M = 4.8, SD = 0.4) and they would recommend the training to other sites (GAT M = 4.5, SD = 0.7; Connector M = 4.9, SD = 0.3).

#### Supervisor training—feasibility, acceptability, and effectiveness

Supervisor training was completed by 117 supervisors across the four sites. Following the training supervisors had a significantly improved understanding of mental health, were significantly more confident that they could identify mental health difficulties in the workplace, recommend professional support, and have a conversation about mental health with their staff (Table 2).

**Table 2:** Paired sample *t*-test comparing mean pre and post impact of supervisor training (*n *= 117)

	Pre mean (SD)	Post mean (SD)	*p*
I am confident that I can describe the difference between mental health, mental health problems and mental illness	2.94 (0.87)	3.92 (0.71)	0.005
I am confident that I can identify if someone was experiencing mental health difficulties in the workplace	3.18 (0.75)	3.82 (0.67)	< 0.001
I am willing to start a conversation with a workmate about their mental health or my concerns for them	3.81 (1.07)	4.16 (0.79)	< 0.001
I am confident that I can identify supports that can be recommended to a person experiencing mental health problems	3.69 (0.99)	4.23 (0.70)	< 0.001
I am confident that I can have an effective conversation about performance issues that may be due to mental health problems	3.31 (0.93)	3.99 (0.74)	< 0.001

Scores from 1 (not at all) to 5 (very much)

### Perceptions of training

The content and delivery of the training was also well received, participants mean scores indicated that they felt that the content was relevant (M = 4.4; SD = 0.7), useful (M = 4.4; SD = 0.7), delivered satisfactorily (M = 4.5; SD = 0.6) and that they would recommend the training to other sites (M = 4.5; SD = 0.7).

#### Outcome analysis

All of the eight mines that participated in Phase One were approached for Phase Two data collection. One mine was unable to participate in Phase Two data collection due to significant changes at the site. The final sample of mines where Phase Two data were collected consisted of sites from both NSW (*n *= 4) and Qld (*n *= 3), and involved open cut (*n *= 3) and underground mining (*n *= 4).

A total of 2508 participants completed the survey across Phase One (*n *= 1457) and Phase Two (*n *= 1051). For sites that participated in the WWMHP, 969 participants completed Phase One (mean site response rate: 76%) with 637 participants at Phase Two (mean site response rate: 86%). At comparator sites, 488 participants completed Phase One (mean site response rate: 60%), and 413 completed Phase Two (mean site response rate: 81%). Key sample demographic and workplace characteristics are provided in Table 3.

**Table 3:** Summary statistics of the WWMHP and control samples across both phases

Personal variables	Control n (%)			WWMHP n (%)		
	Phase 1	Phase 2	*p*	Phase 1	Phase 2	*p*
Sex						
Male	425 (87.6)	379 (92.2)	0.208	841 (87.4)	544 (87.6)	0.358
Female	60 (12.4)	32 (7.8)		121 (12.6)	77 (12.4)	
Age						
< 24	38 (7.8)	24 (5.8)	0.814	78 (8.1)	54 (8.5)	0.953
25–34	143 (29.4)	117 (28.3)		305 (31.8)	188 (29.6)	
35–44	141 (29.0)	128 (31.0)		305 (31.8)	207 (32.6)	
45–54	124 (25.5)	108 (26.2)		207 (21.6)	139 (21.9)	
55+	40 (8.2)	36 (8.7)		65 (6.8)	47 (7.4)	
Relationship status						
Not married or de facto	81 (16.7)	54 (13.1)	0.757	124 (12.9)	92 (14.6)	0.539
Married or de facto	373 (76.7)	327 (79.4)		779 (81.2)	501 (79.3)	
Separated/divorced/widowed	32 (6.6)	31 (7.5)		56 (5.8)	39 (6.2)	
Mine type						
Open cut	174 (35.7)	206 (49.9)	0.039	599 (61.8)	347 (54.5)	0.242
Underground	314 (64.3)	207 (50.1)		370 (38.2)	290 (45.5)	
Mine workers						
FIFO/DIDO	303 (62.2)	342 (83.2)	< 0.001	111 (11.5)	71 (11.2)	0.370
Local	184 (37.8)	69 (16.8)		856 (88.5)	565 (88.8)	
Employment category						
Manager	26 (5.3)	16 (3.9)	< 0.001	42 (4.3)	16 (2.5)	< 0.001
Professional	85 (17.5)	28 (6.8)		113 (11.7)	22 (3.5)	
Trades worker	179 (36.8)	147 (35.9)		315 (32.5)	189 (29.9)	
Machinery operator	155 (31.8)	192 (46.8)		452 (46.6)	384 (60.7)	
Admin or other	42 (8.6)	27 (6.6)		47 (4.9)	22 (3.5)	

Logistic regression was used to examine differences in the composition of the sample between Phase One and Phase Two, across both treatment conditions

Of the 1051 participants who completed the Phase Two survey, 588 (55.9%) indicated that they had participated in the Phase One survey. Measurement of individual change was possible for 332 (56%) participants who completed both Phases and provided matching codes.

Of the 637 participants from sites that received the WWMHP at Phase Two, 329 (51.6%) indicated that they had participated in at least one of the WWMHP programs, with 305 (47.9%) completing GAT, 37 (5.8%) self-reporting Connector training, and 12 (1.9%) completing ASIST (note: some participated in more than one type of training).

#### Mental illness stigma

Stigma was measured by participants indicating whether they felt an employee experiencing mental illness would be ‘treated poorly in the workplace if people found out about it’. The findings indicate a trend towards a decrease in stigma across both control and WWMHP sites, however the effect of time or treatment was not significant (*p *> 0.01) (see Table 4).

**Table 4:** Mental illness stigma by mental health program %

	Low stigma (%)	Unsure (%)	High stigma (%)	*p*
Control				
Phase One	43.8	33.7	22.5	0.360
Phase Two	48.6	31.1	20.3	
MH program				
Phase One	51.8	27.9	20.2	0.284
Phase Two	56.0	25.7	18.4	

#### Responses to mental health problems

The proportion of participants who reported seeking assistance for mental health needs, either professional or non-professional help, was high. The GP was the most common professional source of support (comparator: Phase One = 17.7%, Phase Two = 17.3%; WWMHP: Phase One = 19.8%, Phase Two = 16.5%), with non-professional sources (e.g. friends and family) also common (comparator: Phase One = 39.4%, Phase Two = 36.6%; WWMHP: Phase One = 40.9%, Phase Two = 30.6%). The data showed a modest non-significant decrease in help-seeking between Phase One and Phase Two, however, this remained considerably higher than national community data (all *p*s > 0.01).

#### Perceived mine commitment to mental health

**Fig. 1:**
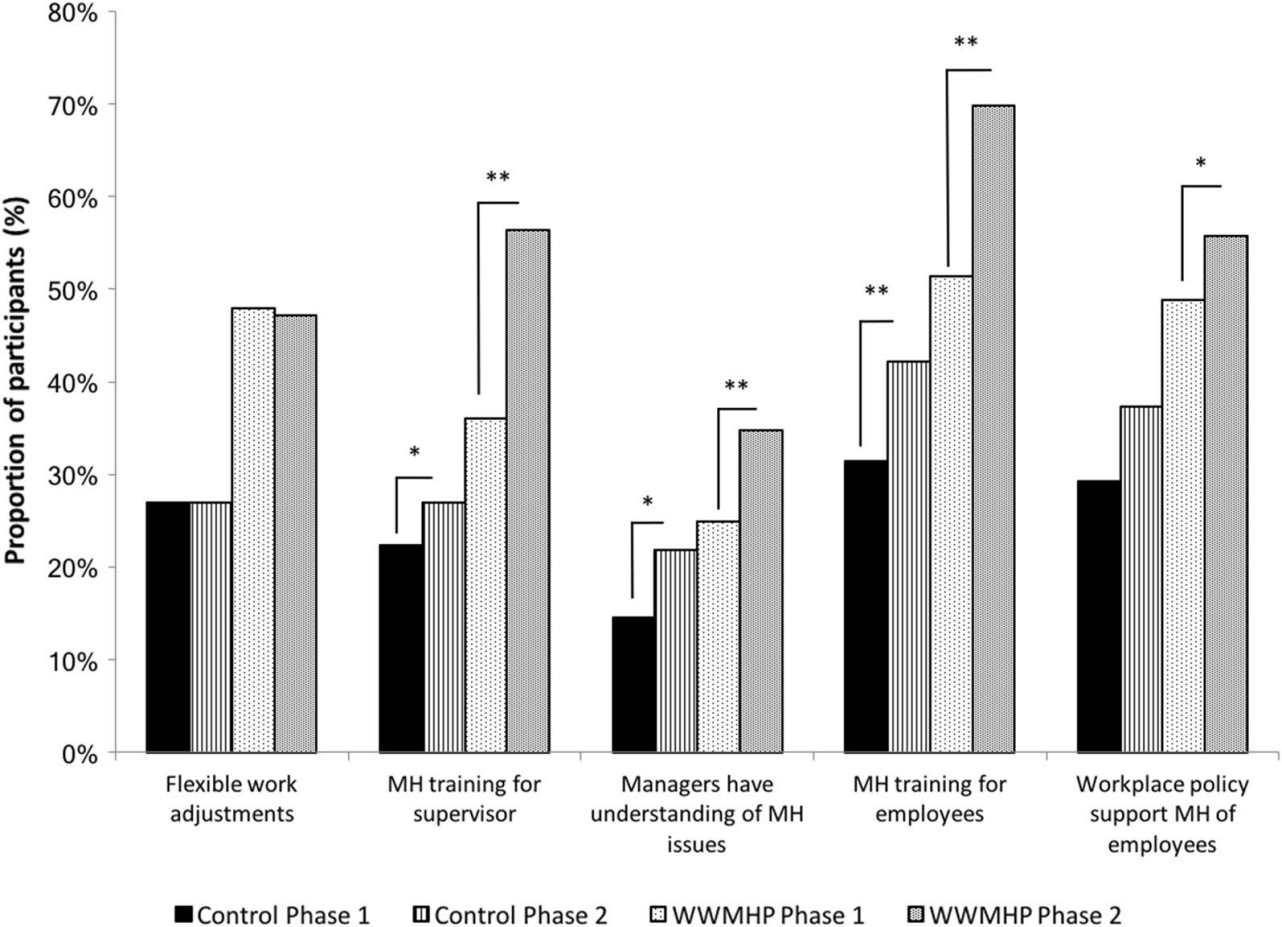
Proportion of employees who perceived mine was committed to employee mental health. *p < 0.01; **p < 0.001

There were a number of significant differences in the perception of the workplace commitment to mental health between Phase One and Phase Two. As shown in Fig. 1, at Phase Two for both WWMHP and control sites there was a significant increase in the proportion of employees who felt that their mine provides education to supervisors (*p *< 0.01) and employees (*p *< 0.01) regarding mental health, and that the managers have a good understanding of mental health related issues (*p *< 0.01). Of note, there was a significant interaction observed between treatment and phase with regards to the perception that mine provides education to supervisors *F*(1,2358) = 3.303, *p *= 0.049, indicating the magnitude of the increase was greater at WWMHP sites. While there was a trend towards significance, there was no significant phase by treatment interaction for perception of the mine providing education to its employees, or that the managers have a good understanding of mental health, despite main effects of time, indicating that there were similar improvements in these measures across both WWMHP and comparator sites. Sites that received the WWMHP did have a significant increase in the proportion of employees who felt that their workplace policies support the mental health of mine employees at Phase Two (*p *< 0.01), a change that was not observed at control sites.

## Discussion

Improving understanding of mental health problems, overcoming stigma, promoting seeking advice and support from available services are important steps in addressing mental ill-health in the workplace. Part of the challenge of implementing programs within workplaces, particularly those within male-dominated industry, is ensuring that they meet the specific needs of the employees, and are both feasible and acceptable. Using information from industry to inform the selection of programs that may be most suitable, the current research demonstrated the feasibility and acceptability of implementing a peer-based mental health support program in the Australian coal mining industry.

### Process evaluation of the WWMHP

#### Mates in mining

A primary aim of the current research was to evaluate the feasibility of implementing a mental health program in the Australian mining industry. Our results indicated significant uptake at participating sites, with the number of employees who completed the GAT representing a considerable proportion of the participating sites’ employees. Feasibility was also demonstrated by the method of implementation, with the GAT often embedded as a component of the sites routine training days. Together, the site uptake and ability to include the program within existing workplace systems provides evidence to support the feasibility of implementing mental health programs within this industry.

Importantly, given the well-recognised challenges of implementing mental health programs in male-dominated industry [8], the training was also considered acceptable by the participating employees. The majority of participants perceived the content as both useful and relevant, and would recommend the training to other sites. Perhaps the clearest demonstration of acceptability however, was the proportion of employees who voluntarily self-nominated (> 10%) to participate in the connector training. The overwhelming response provided evidence to suggest that the employees were interested in taking a more active role in addressing mental health in their workplace.

The evaluation also provided evidence of immediate effectiveness, with significant changes in employee attitudes. Employees were significantly more confident and felt better equipped to begin a conversation about mental health with a colleague they were concerned about after completing training. Identification of early indicators of mental health problems in the workplace and perceived capacity to intervene are desirable characteristics of effective workplace mental health programs [8]. The current study provided evidence to suggest the effectiveness of the MIM program, an adaptation of a program developed for, and previously evaluated in the construction industry [28–31].

#### Supervisor training

Supervisor training was associated with a number of positive outcomes demonstrating acceptability and feasibility. Overall satisfaction with supervisor training was high, with content identified as both relevant and useful for their role as a supervisor. Supervisors reported significant improvements in mental health literacy, with increased confidence in identifying mental health difficulties in the workplace and capacity to offer support and recommend professional services. Greater confidence in being able to have an effective conversation about performance issues that may be due to a mental health related problem and greater willingness to start a conversation with staff they were managing about their mental health was reported. The self-reported ability to recognise specific signals that might indicate mental ill-health, and awareness of the supports available are important foundations for promoting early assistance for people experiencing mental health problems. Supervisors play a pivotal part in creating a work environment that values and respects workers and cares about their well-being and improving the psychosocial work environment [34, 35]. Our results align with previous studies that indicate providing supervisors with necessary skills and information on mental health, including relevant occupational stressors had a favourable effect on workers mental health [36].

### Outcome evaluation

#### Mental illness stigma

Stigma is often cited as one of the greatest barriers to accessing treatment for mental illness [37]. In the current study, in relation to the perception of workplace mental health stigma, fewer people felt that a person would be treated poorly if they disclosed mental ill-health at Phase Two when compared to Phase One. These findings suggest that an ongoing dialogue about mental health is necessary, and with continued commitment to and promotion of mental health awareness, the cultural stigma associated with mental ill-health is likely continue to decline.

#### Help-seeking for mental ill-health problems

Treatments for mental ill-health are effective but access to treatment is poor. Ensuring that individuals feel confident and aware of how to seek help for mental ill-health can impact upon the treatment of mental illness [38]. Encouragingly, this study showed that many employees self-reported discussing their own mental health with professional and non-professional sources of support. Despite having a predominantly male sample, the overall proportion of participants who reported professional service use was almost double the national estimate from the NSMHWB (11.9%). It is unclear why service utilisation was higher than reported previously. One possibility is that the high proportion of service use reflected a greater need for accessing services, which is supported by the higher levels of psychological distress observed in the current study than in the NSMHWB. Nevertheless, the results show that a considerable proportion of coal miners are actively engaged in activities to improve their mental health.

#### Perceived mine commitment to mental health

Participation in the WWMHP created a significant positive change in the employees’ perception of their workplace commitment to the mental health of its employees. There was a significant increase in the proportion of employees who felt the workplace provided education to employees and supervisors about mental health, and a corresponding significant increase in the perception that the mine management have a good understanding of mental health related issues. Interestingly, a subset of these improvements were also observed at control sites, which may indicate that minimal intervention (e.g. a survey on mental health) may have generated interest in, and improved awareness of mental health related issues. While we did not observe a change in the perception of employees around the offering of flexible work adjustments, the results do show that participation in the research has created an environment where employees feel more aware of mental health related issues, and perceive their workplace as committed to the mental health of its employees. This is consistent with other studies that have shown workplace commitment to mental health using a range of strategies, including education, addressing individual workplace risk factors and ensuring supports available for those experiencing a mental illness are effective [4, 18, 39, 40].

## Strengths and limitations

In implementing and conducting a workplace mental health program, several lessons have been learned regarding the contextual influences related to the mining industry. The study was conducted throughout a time of significant economic change in the industry, with resultant organisational restructuring and staffing changes which may have impacted upon employee mental health. One of the control sites elected to discontinue their participation in the research. However, participant involvement and engagement in the study was strong, supporting the need to address mental health problems within the industry. Internal mining champions who promoted participation in the program at a site level were vital to the success of the WWMHP and are seen as important to assist acceptance of mental health programs within the workplace.

The process evaluation measured participant perceptions and attitudes, however we are unable to determine if this translates into changes in participant behaviour. Future research could also investigate whether these changes in attitudes are sustained over time, or whether refresher courses are needed to induce long-term change.

Completing research within an applied and real-life industry setting naturally encounters challenges related to the competing interests of all involved, including tensions related to the  allocation of time to participate and productivity. As such, access to workers for research data collection and to attend the WWMHP required all parties to be dedicated, flexible and responsive. Training days, which regularly occur at some mines, were an ideal time to approach participants for components of this study.

## Conclusion

The WWMHP project included: a general awareness education program and peer assisted model of support; and supervisor/leader training, which built employee resilience through improved coping skills and social support; promoted better understanding of common mental health problems and effective ways of responding if concerned about oneself or someone else. The WWMHP promoted a healthy workplace culture including stigma reduction, while promoting support among work teams and groups. This program was well received by employees, supervisors and managers. The success of this mental health program is the result of a combination of evidence-based programs and services, embedded within workplace policy and undertaken with a robust organisation-wide strategic approach.

## Abbreviations

ASIST: applied suicide intervention skills training
gat: general awareness training
MIM: MATES in mining
NSMHWB: National Survey of Mental Health and Wellbeing
WWMHP: working well mental health program

## References

[1] Slade J, Teesson W, Burgess P. The mental health of Australians 2: report on the 2007 national survey of mental health and wellbeing. 2009.

[2] Australian Bureau of Statistics (2008). National survey of mental health and wellbeing 2007: summary of results.

[3] Beyondblue (2014). Creating a mentally healthy workplace: return on investment analysis.

[4] Harvey S, Joyce S, Tan L, Johnson A, Nguyen H, Modini M, Groth M (2014). Developing a mentally healthy workplace: a review of the literature—a report for the national mental health commission and the mentally healthy workplace alliance.

[5] Cancelliere C, Cassidy JD, Ammendolia C, Côté P (2011). Are workplace health promotion programs effective at improving presenteeism in workers? A systematic review and best evidence synthesis of the literature. BMC Public Health.

[6] PricewaterhouseCoopers (2014). Creating a mentally healthy workplace: return on investment analysis.

[7] Laplagne P, Glover M, Shomos A (2007). Effects of health and education on labour force participation, staff working paper.

[8] Roche AM, Pidd K, Fischer JA, Lee N, Scarfe A, Kostadinov V (2016). Men, work, and mental health: a systematic review of depression in male-dominated industries and occupations. Saf Health Work.

[9] Cotton SM, Wright A, Harris MG, Jorm AF, McGorry PD (2006). Influence of gender on mental health literacy in young Australians. Aust N Z J Psychiatry.

[10] Tynan RJ, Considine R, Rich JL, Skehan J, Wiggers J, Lewin TJ, James C, Inder K, Baker AL, Kay-Lambkin F (2016). Help-seeking for mental health problems by employees in the Australian mining industry. BMC Health Serv Res.

[11] Reavley NJ, Jorm AF (2011). Stigmatizing attitudes towards people with mental disorders: findings from an Australian national survey of mental health literacy and stigma. Aust N Z J Psychiatry.

[12] Corrigan P (2004). How stigma interferes with mental health care. Am Psychol.

[13] Clement S, Schauman O, Graham T, Maggioni F, Evans-Lacko S, Bezborodovs N, Morgan C, Rüsch N, Brown J, Thornicroft G (2015). What is the impact of mental health-related stigma on help-seeking? A systematic review of quantitative and qualitative studies. Psychol Med.

[14] Milner A, Spittal MJ, Pirkis J, LaMontagne AD (2013). Suicide by occupation: systematic review and meta-analysis. Br J Psychiatry.

[15] Australian Bureau of Statistics Causes of Death, Australia. http://www.abs.gov.au/ausstats/abs@.nsf/Lookup/by%20Subject/3303.0~2013~Main%20Features~Suicides~10004. 2013.

[16] Ames G, Bennett JB (2011). Prevention interventions of alcohol problems in the workplace: a review and guiding framework. Alcohol Res Health.

[17] Mills PR, Kessler RC, Cooper J, Sullivan S (2007). Impact of a health promotion program on employee health risks and work productivity. Am J Health Promot.

[18] Dietrich S, Deckert S, Ceynowa M, Hegerl U, Stengler K (2012). Depression in the workplace: a systematic review of evidence-based prevention strategies. Int Arch Occup Environ Health..

[19] McKernon S, Allen R, Money E, Morrow L, Verins I, Willis E (2002). Mentally healthy workplaces—a living toolkit. Mental health and work: issues and perspectives.

[20] Jacobs G (2015). The impact of FIFO work practices on mental health. Final report.

[21] Iversen A, van Staden L, Hughes J, Greenberg N, Hotopf M, Rona R, Thornicroft G, Wessely S, Fear N (2011). The stigma of mental health problems and other barriers to care in the UK Armed Forces. BMC Health Serv Res.

[22] Langston V, Greenberg N, Fear N, Iversen A, French C, Wessely S (2010). Stigma and mental health in the Royal Navy: a mixed methods paper. J Mental Health.

[23] Humphreys JS, Mathews-Cowey S, Weinand HC (1997). Factors in accessibility of general practice in rural Australia. Med J Aust.

[24] Tynan RJ, Considine R, Wiggers J, Lewin TJ, James C, Inder K, Kay-Lambkin F, Baker AL, Skehan J, Perkins D (2016). Alcohol consumption in the Australian coal mining industry. Occup Environ Med..

[25] Considine R, Tynan R, James C, Wiggers J, Lewin T, Inder K, Perkins D, Handley T, Kelly B (2017). The contribution of individual, social and work characteristics to employee mental health in a coal mining industry population. PLoS ONE.

[26] McLean KN (2012). Mental health and well-being in resident mine workers: out of the fly-in fly-out box. Aust J Rural Health.

[27] Torkington AM, Larkins S, Gupta TS (2011). The psychosocial impacts of fly-in fly-out and drive-in drive-out mining on mining employees: a qualitative study. Aust J Rural Health.

[28] Gullestrup J, Lequertier B, Martin G (2011). MATES in construction: impact of a multimodal, community-based program for suicide prevention in the construction industry. Int J Environ Res Public Health.

[29] Martin G, Gullestrup J, Lester D, Gunn JF, Quinnett P (2014). Help-seeking in men: an innovative suicide prevention program form the construction industry. Suicide in men.

[30] Martin G, Swannwell S, Milner A, Gullestrup J (2016). Mates in construction suicide prevention program: a five year review. J Community Med Health Educ.

[31] Doran CM, Ling R, Gullestrup J, Swannell S, Milner A (2016). The impact of a suicide prevention strategy on reducing the economic cost of suicide in the New South Wales construction industry. Crisis.

[32] Dollard M, Bailey T, McLinton S, Richards P, McTernan W, Taylor A, Bond S. The Australian workplace barometer: report on psychosocial safety climate and worker health in Australia. Magill, South Australia: University of South Australia, Centre for Applied Psychological Research for Safe Work Australia; 2012.

[33] Kelly BJ, Stain HJ, Coleman C, Perkins D, Fragar L, Fuller J, Lewin TJ, Lyle D, Carr VJ, Wilson JM, Beard JR (2010). Mental health and well-being within rural communities: the Australian rural mental health study. Aust J Rural Health.

[34] Michie S, Williams S (2003). Reducing work related psychological ill health and sickness absence: a systematic literature review. Occup Environ Med.

[35] Burton J, World Health Organization. WHO Healthy workplace framework and model: background and supporting literature and practices. 2010.

[36] Tsutsumi A (2011). Development of an evidence-based guideline for supervisor training in promoting mental health: literature review. J Occup Health.

[37] Brohan E, Slade M, Clement S, Thornicroft G (2010). Experiences of mental illness stigma, prejudice and discrimination: a review of measures. BMC Health Serv Res.

[38] Bilsker D, Gilbert M, Larry Myetter T, Stewart-Patterson C (2006). Depression and work function: bridging the gap between mental health care and the workplace.

[39] Montano D, Hoven H, Siegrist J (2014). Effects of organisational-level interventions at work on employees’ health: a systematic review. BMC Public Health.

[40] LaMontagne A, Martin A, Page K, Reavley N, Noblet A, Milner A, Keegel T, Smith P (2014). Workplace mental health: developing an integrated intervention approach. BMC Psychiatry.

